# Transcriptome analysis of normal-appearing white matter reveals cortisol- and disease-associated gene expression profiles in multiple sclerosis

**DOI:** 10.1186/s40478-019-0705-7

**Published:** 2019-04-25

**Authors:** Jeroen Melief, Marie Orre, Koen Bossers, Corbert G. van Eden, Karianne G. Schuurman, Matthew R. J. Mason, Joost Verhaagen, Jörg Hamann, Inge Huitinga

**Affiliations:** 1 Department of Neuroimmunology, Netherlands Institute for Neuroscience, Institute of the Royal Netherlands Academy of Arts and Sciences, Amsterdam, The Netherlands; 2 Department of Astrocyte Biology and Neurodegeneration, Netherlands Institute for Neuroscience, Institute of the Royal Netherlands Academy of Arts and Sciences, Amsterdam, The Netherlands; 3 Department of Neuroregeneration, Netherlands Institute for Neuroscience, Institute of the Royal Netherlands Academy of Arts and Sciences, Amsterdam, The Netherlands; 4 Department of Experimental Immunology, Amsterdam Infection & Immunity Institute, Amsterdam UMC, University of Amsterdam, Amsterdam, The Netherlands

**Keywords:** Multiple sclerosis, Normal-appearing white matter, HPA axis, Transcriptome

## Abstract

Inter-individual differences in cortisol production by the hypothalamus–pituitary–adrenal (HPA) axis are thought to contribute to clinical and pathological heterogeneity of multiple sclerosis (MS). At the same time, accumulating evidence indicates that MS pathogenesis may originate in the normal-appearing white matter (NAWM). Therefore, we performed a genome-wide transcriptional analysis, by Agilent microarray, of post-mortem NAWM of 9 control subjects and 18 MS patients to investigate to what extent gene expression reflects disease heterogeneity and HPA-axis activity. Activity of the HPA axis was determined by cortisol levels in cerebrospinal fluid and by numbers of corticotropin-releasing neurons in the hypothalamus, while duration of MS and time to EDSS6 served as indicator of disease severity. Applying weighted gene co-expression network analysis led to the identification of a range of gene modules with highly similar co-expression patterns that strongly correlated with various indicators of HPA-axis activity and/or severity of MS. Interestingly, molecular profiles associated with relatively mild MS and high HPA-axis activity were characterized by increased expression of genes that actively regulate inflammation and by molecules involved in myelination, anti-oxidative mechanism, and neuroprotection. Additionally, group-wise comparisons of gene expression in white matter from control subjects and NAWM from (subpopulations of) MS patients uncovered disease-associated gene expression as well as strongly up- or downregulated genes in patients with relatively benign MS and/or high HPA-axis activity, with many differentially expressed genes being previously undescribed in the context of MS. Overall, the data suggest that HPA-axis activity strongly impacts on molecular mechanisms in NAWM of MS patients, but partly also independently of disease severity.

**Electronic supplementary material:**

The online version of this article (10.1186/s40478-019-0705-7) contains supplementary material, which is available to authorized users.

## Introduction

Multiple sclerosis (MS) is a chronic neurodegenerative disease that is hallmarked by the presence of inflammatory demyelinating lesions in the central nervous system (CNS). Therefore, major efforts have been dedicated to identify the molecular mechanisms at play during formation and expansion of MS lesions [45, 51, 53]. Related to this, there is an increasing interest for normal-appearing white matter (NAWM) as starting point of MS pathogenesis, as NAWM in MS was found to contain numerous cellular and molecular changes in inflammatory and neuroprotective pathways [60, 82, 89]. Studying these pathways may lead to the identification of events that precede or determine permissiveness for development of new MS lesions [16].

Clinical and pathological heterogeneity is one of the most striking features of MS [5, 53, 54, 84]. A factor considered to be associated with heterogeneity of MS is activity of the hypothalamus–pituitary–adrenal (HPA) axis [25, 26, 32, 58, 75]. Recently, we found in a post-mortem study of 42 MS patients that high levels of cortisol in the cerebrospinal fluid (CSF) were associated with slow disease progression, in particular in females with secondary-progressive MS, whereas patients with low cortisol levels had fast disease progression, greater numbers of active lesions, and less remyelinated plaques [58]. Moreover, NAWM of female secondary-progressive MS patients with high cortisol levels displayed elevated expression of glucocorticoid-responsive genes, such as CD163, and decreased expression of pro-inflammatory genes, such as tumor necrosis factor, when compared to NAWM of patients with low cortisol levels. These data strongly suggest that high HPA-axis activity, by means of cortisol hypersecretion, impacts on molecular mechanisms in the NAWM in such a way that it reduces permissiveness for lesion development. Defining the molecular pathways induced in NAWM by cortisol may therefore identify targets for the development of therapeutic strategies to slow down or halt MS progression.

The heterogeneity of MS has been established at the clinical, radiological, and neuropathological level, but not with regard to molecular mechanisms in NAWM [5, 53, 54]. Without addressing disease heterogeneity, an earlier study on genome-wide expression in MS NAWM showed an upregulation of genes involved in anti-inflammatory mechanisms in oligodendrocytes and increased mRNA levels of pro-inflammatory molecules [89]. Thus, no study thus far addressed the question whether and to what extent gene expression profiles in NAWM of MS patients reflect disease severity and/or HPA-axis activity. Here, we applied an Agilent microarray to perform a genome-wide transcriptional analysis of post-mortem NAWM of 18 female subjects with secondary-progressive MS that displayed a strong heterogeneity in disease severity and HPA-axis activity. This allowed us to investigate gene expression in relation to disease severity and/or HPA-axis activity. No male subjects were included, as an earlier study by us indicate that the inverse correlation of HPA axis activity with both disease severity and pathology of MS was most prominent in females with secondary progressive MS [58]. As an added benefit, this provided us with a more homogeneous population, excluding possible confounding effects of gender on our findings.

We analyzed our data using various approaches, including WGCNA and group-wise comparisons of control subjects and (subpopulations of) MS patients. The WGCNA enabled us to identify clusters of co-regulated genes that correlate with one or more indicators of MS-disease severity and HPA-axis activity. In addition, individual genes were also studied for their association with the same clinical and endocrinological parameters. This led us to uncover many novel genes positively or negatively associated with HPA-axis activity and/or severity of MS. In general, gene expression profiles associated with high cortisol production and mild MS were characterized by molecules that actively regulate inflammation, but also belong to pathways involved in proliferation of neural stem cells. The data indicate that HPA-axis activity strongly impacts on molecular mechanisms in NAWM of MS patients and strongly affects pathways associated with disease severity. In this way, our study identifies molecular targets that may be assessed for their potential to prevent MS pathology in NAWM or limit lesion formation.

## Materials and methods

### Brain tissue

Snap-frozen tissue from control and MS-brain donors was obtained from the Netherlands Brain Bank, Amsterdam, The Netherlands (http://www.brainbank.nl). Informed consent was obtained for brain autopsy and the use of tissue and clinical information for research purposes. Exclusion criteria were death due to sepsis and glucocorticoid treatment within 8 weeks prior to death. Clinical diagnoses of MS were confirmed by a neurologist (Prof. C.H. Polman, VUmc, Amsterdam or Dr. S. Luchetti, NIN, Amsterdam, The Netherlands). An overview of the pathological and clinical data of the brain donors is depicted in Table 1.

**Table 1:** Overview of included subjects

NBB no.	Sex	Age	PMD	pH	MS/C	Onset	Duration	Time to EDSS6	Type	EDSS	Death cause
96–026	F	69	9:15	6.40	MS	44	25	16	SP	9	respiratory insufficiency
96–074	F	40	7:00	6.74	MS	26	14	11	SP	8–9	dehydration
96–076	F	81	4:15	6.93	MS	32	49	44	SP	6	cachexia
96–121	F	53	7:15	6.54	MS	35	18	8	SP	9	pneumonia
97–006	F	62	6:45	6.49	MS	33	29	22	SP	9	cardiac asthma
97–160	F	40	7:00	6.33	MS	29	11	8	SP	9	aspiration pneumonia with cardiac decompensation
98–009	F	70	6:30	6.30	MS	38	32	n/a	SP	9	cardiac arrest
98–158	F	76	14:15	5.93	MS	23	53	24	SP	9	respiratory insufficiency
99–025	F	64	7:45	6.22	MS	29	35	21	SP	9	pneumonia and dehydration
99–054	F	58	8:10	6.30	MS	38	20	13	SP	9	legal euthanasia
99–073	F	71	8:00	6.80	MS	47	24	30	SP	9	pneumonia
99–086	F	71	10:25	6.35	MS	47	24	22	SP	9	respiratory insufficiency
99–119	F	38	5:15	6.55	MS	28	10	n/a	RR	3	cardiac arrest
00–120	F	69	13:20	6.12	MS	43	26	10	SP	9	probable viral infection
01–018	F	48	8:10	6.55	MS	40	8	7	SP	6.5	legal euthanasia
01–093	F	66	6:20	6.44	MS	23	43	30	SP	9	liver failure due to cancer metastases
01–126	F	80	9:35	6.20	MS	21	59	51	SP	9	acute leukemia
02–053	F	48	5:50	6.64	MS	27	21	14	SP	8	heart failure
95–078	F	74	6:40	6.70	C	–	–	–	–	–	cachexia
96–014	F	54	8:00	6.45	C	–	–	–	–	–	acute renal failure
00–025	F	68	5:45	6.97	C	–	–	–	–	–	legal euthanasia
01–069	F	41	13:30	–	C	–	–	–	–	–	pulmonary artery hemorrhage
97–068	F	61	7:15	7.20	C	–	–	–	–	–	cachexia
97–042	F	65	12:50	6.90	C	–	–	–	–	–	cardiac arrest
97–005	F	69	7:10	9.80	C	–	–	–	–	–	respiratory insufficiency
96–051	F	71	4:50	6.70	C	–	–	–	–	–	cardiac arrest
00–050	F	52	6:30	7.20	C	–	–	–	–	–	metastasized leiomyosarcoma

NBB no. = donor registration number of the Netherlands Brain Bank

*Age* age at death (years), *PMD* post-mortem delay (hours:minutes), *pH* pH of CSF, *MS/C* MS or control subject, *Onset* age of disease onset (years), *Duration* disease duration (years), *Time to EDSS6* time to EDSS6 (years), *Type* clinical subtype of MS, *F* female, *SP* secondary progressive MS, *RR* relapsing-remitting MS, *n/a* not available

### Quantification of CRH-producing neurons and cortisol levels

Numbers of corticotropin-releasing hormone (CRH)-expressing neurons in the paraventricular nucleus (PVN) were quantified in fixed tissue as described previously [35, 58]. In short, serial 6-μm frontal sections were cut on a microtome. Delineation of the PVN was determined in thionine-stained sections. Each 100th section through the PVN was stained for CRH. Neurons that showed a nucleolus and expressed CRH were counted blinded. The total number of CRH-expressing neurons in the PVN was calculated on the basis of cell counts and the distance between the sections. Cortisol was measured by radioimmunoassay (Diagnostic Products Corporation, Los Angeles, CA, USA), using a radioactively labeled antibody that enables highly sensitive detection of cortisol levels in various types of fluid, including serum, CSF, and saliva.

### Tissue processing and RNA isolation

Series of 10 cryostat sections (20 μm each) of subcortical NAWM were homogenized in Trizol (Invitrogen, Carlsbad, CA, USA). Sections preceding and following these series were stained by immunohistochemistry for proteolipid protein (PLP; Serotec, Oxford, UK) and HLA-DP, −DQ, −DR (DakoCytomation, Glostrup, Denmark) to confirm the absence of MS-lesion pathology, respectively, by ruling out demyelination and microglia/macrophage activation. RNA isolation and assessment of its quality by RNA integrity number (RIN) was performed as described previously [40]. Along with this, RNA was extracted from snap-frozen tissue dissected from various anatomical regions of control and MS brains, including MS lesions and NAWM, as well from tonsil. This RNA which was pooled to create common reference complementary RNA (cRNA), which was co-hybridized to every microarray slide to enable accurate comparison of expression levels across different cDNA microarray experiments.

### Microarray hybridization

Labeling of isolated RNA was done using the Low Input Quick Amp Labeling kit (Agilent Technologies, Palo Alto, CA, USA), according to the manufacturer’s instructions. For whole-genome expression analysis, samples were hybridized to Agilent 4x44K v2 Whole Human Genome arrays (G4845A; Agilent Technologies), covering 27,958 genes. In brief, equal amounts of total RNA (50 ng) were amplified and labeled with either Cy3-CTP (experimental samples) or Cy5-CTP (reference material, obtained as described above) using the Low Input Quick Amp Labeling kit (Agilent Technologies). For hybridization, equal amounts (825 ng) of labeled samples were fragmented in Fragmentation Buffer (Agilent Technologies) for 30 min at 60 °C. Labeled and fragmented complementary RNA (cRNA) was hybridized to the array and incubated in a rotating hybridization chamber for 17 h at 60 °C. After hybridization, the array was washed subsequently for 5 min in 6 x saline sodium phosphate-EDTA (SSPE)/0.005% N-lauroylsarcosine, 1 min in 0.006 x SSPE/0.005% N-lauroylsarcosine, and 30 s in acetonitrile and dried quickly in nitrogen flow. The arrays were scanned at a resolution of 5 mm and at 5 and 100% photomultiplier tube settings using the Agilent DNA Microarray Scanner (Agilent Technologies). Scan data were extracted using Agilent Feature Extraction software (version 8.5.1; Agilent Technologies).

### Normalization of gene expression and gene extraction

**Fig. 1:**
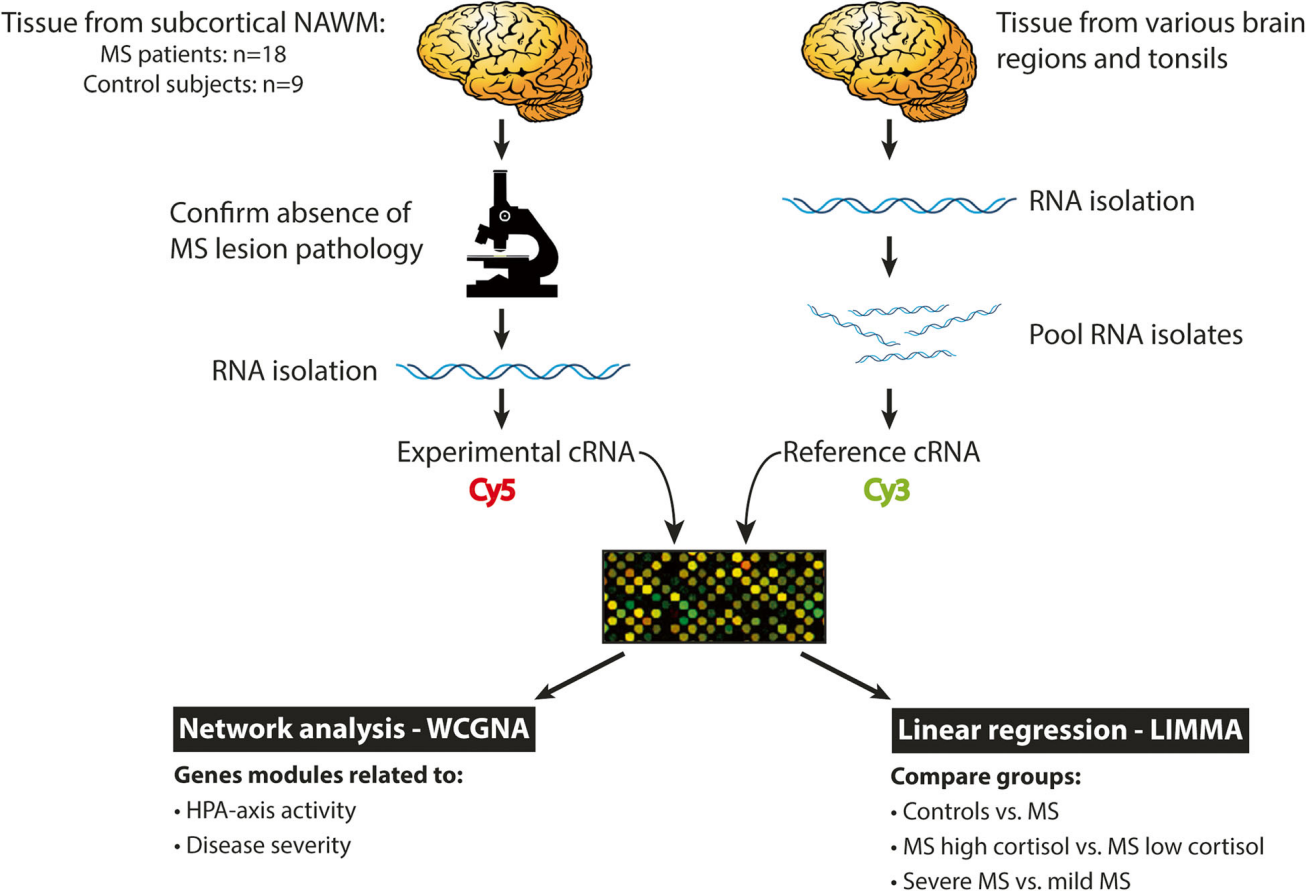
Schematic representation of the experimental approach. Series of cryostat sections of post-mortem brain tissue, dissected from subcortical NAWM of 18 MS patients and 9 control subjects, were used for RNA extraction. Sections preceding and following these series were stained by immunohistochemistry for proteolipid protein and HLA-DP, −DQ, −DR to confirm the absence of MS lesion pathology. In parallel, RNA was extracted from snap-frozen tissue dissected from a diversity of anatomical regions from control and MS brains, including MS lesions and NAWM, as well from tonsil, which was pooled and used to generate common reference cRNA. Common reference cRNA was co-hybridized to every microarray slide to allow for accurate comparison of expression levels across different cDNA microarray experiments. In this way, a ratio between the experimental and reference material could be calculated for every spot, and expression levels across different hybridizations could be compared. These data were subjected to WGCNA to identify clusters of co-regulated genes associated with HPA-axis activity and severity of MS. Furthermore, the data were used for group-wise comparisons between control subjects and MS patients subdivided into subgroups with high and low cortisol levels or subgroups with severe and mild disease using LIMMA

To allow for accurate comparison of expression levels across different cDNA microarray experiments, common reference cRNA was co-hybridized to every microarray. By using the reference cRNA, a ratio between the experimental and reference material could be calculated for every spot, and expression levels across different hybridizations were compared. These data were used for analysis by weighted gene co-expression network analysis (WGCNA) and by group-wise comparison between subgroups, which is further described below. For an overview of the experimental setup, see Fig. 1. Raw data from the extraction software was imported to the R statistical processing environment using linear models for microarray data (LIMMA)/Bioconductor (version 3.12.3) package. Feature and background non-uniformity outliers, as determined by the Agilent Feature Extraction software using default settings, were removed. The data were normalized using “between array normalization” with the “Gquantile” algorithm (http://www.bioconductor.org).

### cDNA synthesis and quantitative real-time PCR

Using the same RNA from which cRNA was generated for hybridization, cDNA was synthesized with the Quantitect Reverse Transcription Kit (Qiagen, Hilden, Germany) according to the manufacturer’s protocol. Quantitative real-time polymerase chain reaction (PCR) was performed and analyzed as described elsewhere, with minor adaptations [40]. The amount of cDNA used per reaction was based on an input of 5 ng original RNA in a final volume of 20 μl.

### Statistical analysis

To obtain lists of genes associated with either disease severity or HPA-axis activity, a WGCNA was performed on the ^2^log intensities to find gene modules [44, 61]. WCGNA defines, in an unbiased way, clusters of genes that show a co-expression pattern amongst samples, creating (a network with) modules of genes that are co-regulated. A soft power threshold of 9 was determined in the recommended way [44], a signed network was used, and dendrogram cluster detection was performed using a minimum cluster size of 27 and a deepSplit value of 1.

In addition to the WCGNA, linear regression analysis was performed for group-wise comparisons, using the LIMMA/bioconductor package in R (version 3.12.3). This was carried out using the limma function “lmscFit” for single channel analysis on two-color arrays with correction for intraspot correlation. A false discovery rate of 0.05 was used by multiple testing correction with the Benjamini-Hochberg method. All models were pairwise comparisons of two conditions. These group-wise comparisons were made for gene expression between all MS patients and control subjects. Furthermore, the group of MS patients was separated into a high and a low cortisol subgroup by the median for this parameter for objective group-wise comparison to identify cortisol-associated gene expression. To perform unbiased group-wise comparisons to find gene expression profiles associated with severity of MS, the patient population was divided into two subgroups by the median disease duration. Correlation coefficients were interpreted as follows: r < 0.1: no correlation; 0.1 < r < 0.3: weak correlation; 0.3 < r < 0.5: moderate correlation; r > 0.5: strong correlation. The significance level was set to 0.05. For all analyses that did not include the microarray data, we used SPSS software version 24 (IBM, Armonk, NY, USA).

All modules identified by WGCNA were subjected to the online tool DAVID, version 6.7 (http://david.abcc.ncifcrf.gov) for functional gene ontology (GO) analysis, using the whole human genome as background. For enrichment of top GO classes, the significance level was set to 0.05 (unadjusted). The minimum number of genes in each GO term was 3.

## Results

Post-mortem NAWM of 18 female subjects with secondary-progressive MS that displayed a strong heterogeneity in disease severity and HPA-axis activity and white matter of 10 matched control subjects was analyzed for differences in gene expression using Agilent Human Gene Expression 4x44K v2 microarrays. Table 2 provides an overview of the brain donor characteristics, including age, post-mortem delay (PMD), pH value of the CSF, and quality of RNA from NAWM in control subjects and different subgroups of MS patients. This table also displays clinical characteristics of the MS patients, such as disease duration and cortisol levels in CSF. No differences were present between control subjects and MS patients in age, PMD, or RIN. However, pH was significantly lower in MS patients compared to controls (*p* = 0.001). At the same time, pH and PMD did not correlate with RIN, cortisol levels, or numbers of CRH-expressing neurons, indicating that they had no confounding effects. There was a significant correlation between cortisol and numbers of CRH-positive neurons in MS patients (r = 0.508, *p* = 0.031), corroborating the idea that indicators of HPA-axis activity measured after death reliably reflect the situation during life [58]. In addition, cortisol levels in CSF correlated with duration of MS (r = 0.490, *p* = 0.039) and with a trend towards significance with time to a score of 6 on the expanded disability status scale (EDSS6) (r = 0.432, *p* = 0.095), confirming earlier findings that high HPA-axis activity coincides with slower disease progression and relatively mild MS [35, 58].

**Table 2:** Overview of parameters used to match control subjects and (subgroups of) MS patients

	Controls (*n* = 9)		All MS patients (*n* = 18)		MS Low cortisol (*n* = 9)		MS High cortisol (*n* = 9)		MS Severe disease (*n* = 10)		MS Mild disease (*n* = 8)	
	Med	IQR	Med	IQR	Med	IQR	Med	IQR	Med	IQR	Med	IQR
Age	66.5	53.0–70.0	65	48–71	58	44–69	70	62–71	51	40–70	70	65–79
Duration	–	–	24	17–37	20	11–31	29	21–48	19	11–22	39	30–52
Cortisol	–	–	221	86–492	86	20–178.0	492	289–669	146	29–461	242	183–639
PMD	7:10	6:00–10:25	7:30	6:25–9:20	7:45	5:30–8:45	7:15	6:45–10:00	7:40	6:40–8:25	7:15	6:20–12:25
pH CSF	7.0	6.7–7.1	6.4	6.3–6.6	6.4	6.3–6.6	6.4	6.3–6.6	6.5	6.3–6.7	6.3	6.1–6.5
RIN	7.4	7.1–8.3	7.8	7.0–8.3	7.7	7.0–8.4	7.4	6.6–7.9	7.6	7.1–8.3	7.7	6.9–8.4

*Med* median, *IQR* interquartile range, *Age* age at death (years), *Duration* disease duration (years), *Cortisol* cortisol levels in CSF (nmol/l), *PMD* post-mortem delay (hours:minutes), *pH CSF* pH value of CSF, *RIN* RNA integrity number

### Weighted gene co-expression network analysis (WGCNA)

A first analysis on the microarray data was done by WGCNA, which in an unbiased way identifies clusters of genes that display a similar expression pattern across the different samples in the whole data set; clusters of genes that show the same expression pattern are referred to as modules. WGCNA can also be used to investigate whether such modules are related to traits of particular interest. Each module has an eigengene (the first principal component) that represents the average expression pattern of all genes within the module. Moreover, for each gene within a module, a correlation value to the module eigengene is calculated, which indicates to what extent the expression profile of an individual gene resembles that of the whole module [44, 61].

Two approaches were taken for WGCNA. The main analysis was performed on a dataset that included the samples from both control subjects and MS patients. Another analysis was performed on data from MS patients only, which was done to confirm findings made in the first approach and to identify additional gene expression profiles associated with HPA-axis activity and disease severity in MS. From both datasets, we selected out of a total of 27,958 genes covered by the microarray the 15,000 genes that showed the biggest variance across all samples for application of WGCNA. By hierarchical clustering using these data we found two outliers, one in the control group and one in the MS patient group (donor 01–069 and 98–158 respectively), which were excluded from further analyses. All data described below in the text, tables, and figures were generated by our main WGCNA analysis that included both control subjects and MS patients, unless stated otherwise. To find gene clusters associated with disease severity and HPA-axis activity in MS, we studied the correlation between gene modules identified by WCGNA and several clinical traits: disease duration and time to EDSS6 as parameters of disease severity, CSF cortisol levels and numbers of CRH-producing neurons as indicators of HPA-axis activity.

### Modules generated by WCGNA correlate to HPA-axis activity and MS severity

**Fig. 2:**
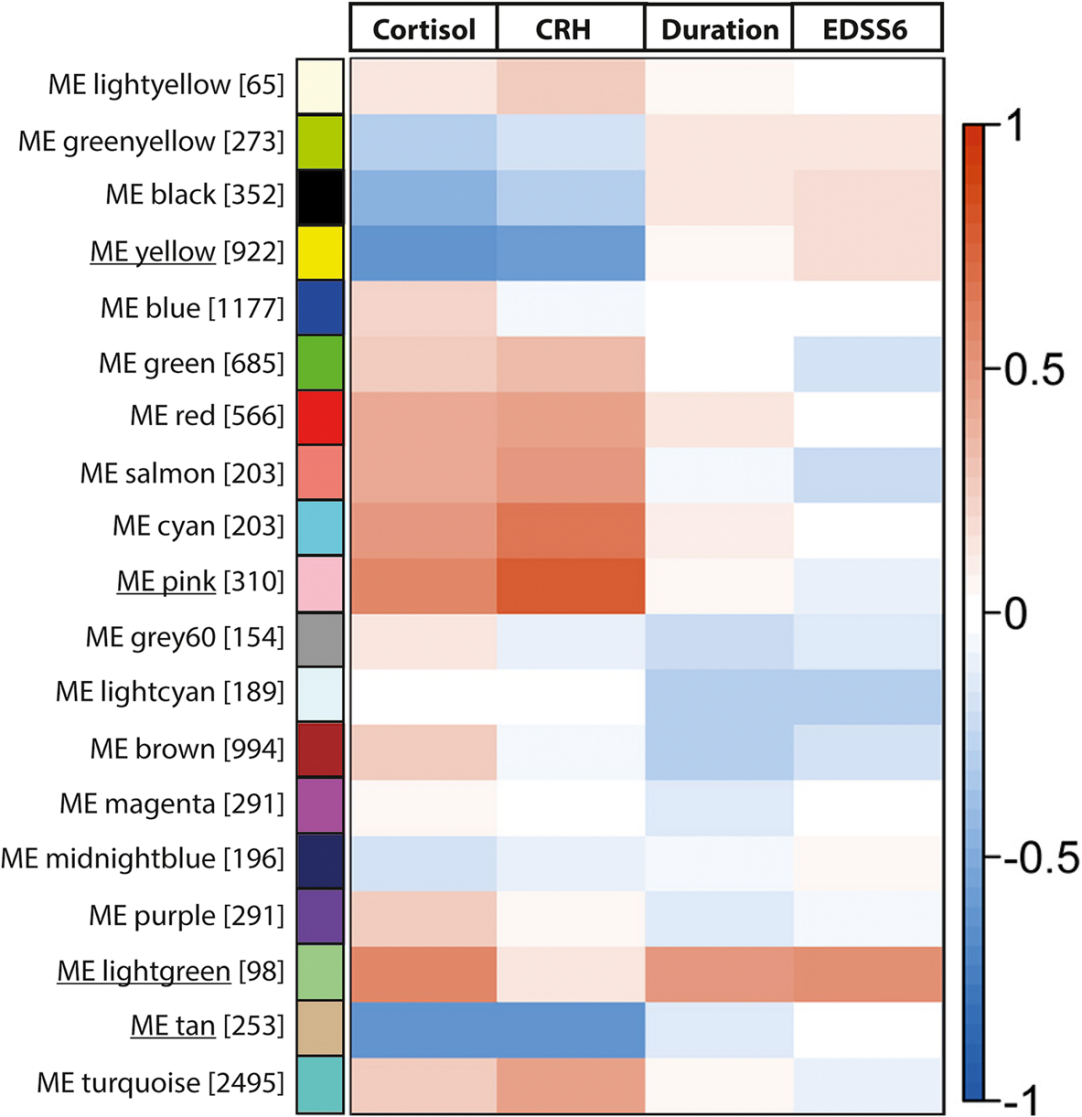
Module trait relationships. Overview of the modules generated by the WCGNA and their relationship with parameters for HPA-axis activity and disease severity. On the left are the names of the modules, the digits between square brackets indicating the number of genes present. The scale on the right indicates the actual values belonging to the coefficients of correlations between the module eigengenes and the studied traits. Underlined are the modules with strongest positive (lightgreen and pink) and negative (tan and yellow) correlations to one or more traits, which were therefore further analyzed and described in the text. Note the positive correlation of the lightgreen module with CSF cortisol levels, disease duration and time to EDSS6. ME = module eigengene; Cortisol = cortisol level in cerebrospinal fluid; CRH = number of neurons expressing corticotropin-releasing hormone; Duration = disease duration; EDSS6 = time to EDSS6

Using the WGCNA method, we identified a total of 19 modules, of which the biggest module contained 2495 genes and the smallest 65 genes (Fig. 2). Of the identified modules, 9 showed an expression pattern that correlated with one or more of the traits of interest: cortisol levels in CSF, number of CRH-positive neurons, disease duration, and time to EDSS6. Notably, all modules that were correlated to CSF cortisol levels also showed a correlation in the same direction to numbers of CRH-expressing neurons, substantiating their association with activity of the HPA axis. In total, eight modules correlated only to indicators of HPA-axis activity, i.e. cortisol levels in CSF and numbers of CRH-expressing neurons in the PVN: the black module (r = − 0.47, *p* = 0.020; r = − 0.31, *p* = 0.100, respectively), yellow module (r = − 0.63, *p* < 0.001; r = − 0.57, *p* = 0.003, respectively), red module (r = 0.43, *p* = 0.030; r = 0.46, *p* = 0.020, respectively), salmon module (r = 0.40, *p* = 0.050; r = 0.52, *p* = 0.008, respectively), cyan module (r = 0.48, *p* = 0.010; r = 0.65, p < 0.001, respectively), pink module (r = 0.56, p = 0.003; r = 0.77, p < 0.001, respectively), tan module (r = − 0.63, p < 0.001; r = − 0.61, *p* = 0.001, respectively), and turquoise module (r = 0.25, *p* = 0.200; r = 0.46, p = 0.020, respectively).

Four modules stood out for their particular strong correlation to one or more traits. The first one is the lightgreen module, which positively correlated to cortisol levels, disease duration, and time to EDSS6. Secondly, the pink module showed a strong positive correlation to cortisol levels and to numbers of CRH-expressing neurons. In contrast, the tan and yellow module correlated negatively to cortisol levels and numbers of CRH neurons. The 10 genes most strongly connected to the expression pattern of the four modules (module eigengene) are depicted in Table 3. The GO classes enriched in these four modules according to functional annotation clustering analysis are depicted in Table 4. The results of the GO analysis of all the other modules are shown in Additional file 1.

**Table 3:** Genes with the strongest connectivity to the modules identified by WGCNA

	Lightgreen module				Pink module					Tan module			Yellow module		
	Gene	Corr	P		Gene	Corr	P		Gene	Corr	P		Gene	Corr	P
1	ASPM	0.97	4.2E-15	1	CHORDC1	0.96	2.7E-14	1	MAGED1	0.92	4.8E-11	1	PPP2R3A	0.96	5.7E-14
2	CST7	0.95	1.7E-13	2	CACYBP	0.95	2.9E-13	2	SARS2	0.90	6.5E-10	2	CNP	0.95	1.8E-13
3	DEFA4	0.95	1.1E-12	3	DNAJA4	0.94	1.4E-12	3	PRMT7	0.90	1.2E-09	3	VPS4B	0.95	4.0E-13
4	VSTM1	0.94	1.2E-12	4	HSPH1	0.94	1.6E-12	4	NAT6	0.88	6.4E-09	4	TMEM209	0.95	5.0E-13
5	BPI	0.94	4.2E-12	5	HSPA4L	0.94	1.9E-12	5	NFU1	0.87	1.1E-08	5	ITCH	0.94	1.8E-12
6	DLGAP5	0.93	1.2E-11	6	DNAJB4	0.94	2.1E-12	6	COG1	0.87	1.2E-08	6	ENDOD1	0.94	2.0E-12
7	S100P	0.93	1.6E-11	7	P4HA2	0.94	3.2E-12	7	THNSL1	0.87	1.5E-08	7	PCBP4	0.94	3.1E-12
8	WISP3	0.93	1.7E-11	8	HSPD1	0.93	8.1E-12	8	RTF1	0.87	2.1E-08	8	ATPGD1	0.94	5.6E-12
9	NLRP12	0.93	2.1E-11	9	HSPE1	0.93	9.5E-12	9	STOML1	0.87	2.2E-08	9	CUEDC1	0.94	6.1E-12
10	RGL4	0.93	3.0E-11	10	ACRC	0.93	1.1E-11	10	NIT2	0.87	2.3E-08	10	CA14	0.94	6.2E-12

*Corr* correlation to the principal component of the module, which represents the connectivity of each gene to the module; *P* = *p*-value

**Table 4:** GO classes overrepresented in modules identified by WGCNA

Lightgreen module	
GO class	Genes present
Immune response (GO:0006955)	EXO1, IL18RAP, PRG2, PGLYRP1, CFP, BPI, CST7, SP2, S1PR4, LILRA5, FCN1, CEACAM8, CTSG
Defense response (GO:0006952)	IL18RAP, RNASE3, S100A8, CEBPE, PRG2, S100A9, PGLYRP1, S100A12, AZU1, CFP, BPI, DEFA4, MPO, CTSG
Regulation of cytokine production (GO:0001817)	AZU1, BPI, ELANE, NLRP12, GHRL
Regulation of IL-1b production (GO:0032652)	AZU1, NLRP12, GHRL
Negative regulation of cytokine biosynthetic process (GO:0042036)	ELANE, NLRP12, GHRL
Immune system development (GO:0002520)	EXO1, CCNB2, CEBPE, FLT3, MMP9
Phagocytosis (GO:0006909)	CEBPE, FCN1, ELANE
Pink module	
GO class	Genes present
Regulation of caspase activity (GO:0043281)	FOXL2, ADORA2A, SMAD6, HSPE1, HSPD1, PMAIP1, DNAJB6
Response to unfolded protein (GO:0006986)	HSP90AB1, HSP90AA2, HSP90AA1, HSPA1A, HSPA1B, SERPINH1, HSPA1L, HSPH1, HSPA4L, DNAJA1, HSPA6, HSPB1, HSPA4, HSPE1, DNAJB1, HSPD1, DNAJB4, HSPA8, DNAJB6
Heat shock protein binding (GO:0031072)	FKBP4, PPID, DNAJA1, DNAJB1, DNAJB4, DNAJA4, DNAJB6
Antigen processing and presentation (KEGG pathway hsa04612)	HSPA1L, HSP90AA2, HSP90AB1, HSP90AA1, HSPA6, HSPA4, HSPA1A, HSPA1B, HSPA8
Regulation of lymphocyte activation (GO:0051249)	TRAF2, ADORA2A, TNFSF14, HSPD1, SOD1, IL7R, SART1
Regulation of T cell mediated immunity (GO:0002709)	TRAF2, HSPD1, IL7R
Cellular response to oxidative stress (GO:0034599)	PYCR1, EPAS1, SIRT7, SOD1
Oxidation reduction (GO:0055114)	CTBP2, HTATIP2, HSD17B1, UGDH, SIRT7, CRYZ, SOD1, PYCR1, CYP39A1, PLOD1, KDM2A, P4HA2, NXN, P4HA1, JMJD6, PLOD3, SPR, BCO2
Tan module	
GO class	Genes present
Generation of precursor metabolites and energy (GO:0006091)	ALDOA, NDUFA5, UQCRC1, AIFM3, NDUFB10, ACO2, FDX1, TMX4, CRAT, UQCRFS1, ATP5G3, COX6C, SDHA, ATP6V0C, ATP5C1, SLC25A3, ATPIF1, NDUFS1, MDH1, PYGB
Mitochondrion (GO:0005739)	RNASEL, UQCRC1, FDX1, NIT2, TMX4, HINT2, GCAT, WARS2, BPHL, UQCRFS1, ATP5G3, SFXN5, GOT2, DDX28, NT5M, GPX4, SLC25A3, TIMM23B, ACAD9, NDUFS1, HSD17B8, PDK1, PDK2, NDUFA5, NDUFB10, AIFM3, ACO2, SLC25A5, AMACR, CRAT, SARS2, COX6C, SDHA, NFU1, MRPS18A, ATP5C1, ATPIF1, PRODH
Yellow module	
GO class	Genes present
Lipid biosynthetic process (GO:0008610)	TM7SF2, CYP51A1, ACSS2, PEX7, ELOVL1, FAR1, ELOVL5, DHCR7, SERINC1, PRKAA1, HSD17B3, PCYT1B, SCD5, PCYT2, AGPAT4, GAL3ST1, PLD1, PLP1, LPGAT1, FADS1, FA2H, PIGT, PIGS, CFTR, CERCAM, PIGP, PTGDS, C5ORF4, SMPD1, MVK, IDI1, DEGS1
Steroid metabolic process (GO:0008202)	TM7SF2, SREBF1, OSBPL5, MBTPS2, CYP51A1, CFTR, ABCA2, DHCR7, INSIG1, PRKAA1, MVK, HSD17B3, IDI1, LIPE, VLDLR, CLN6

*GO* gene ontology; *KEGG* Kyoto Encyclopedia of Genes and Genomes

### A module strongly correlated to both severity of MS and HPA-axis activity

Among the gene clusters identified by the WCGNA, as depicted in Fig. 2, the lightgreen module had an eigengene that was positively correlated to cortisol levels (r = 0.56, *p* = 0.003), disease duration (r = 0.50, *p* = 0.010), and time to EDSS6 (r = 0.54, *p* = 0.006), indicating its positive association with both HPA-axis activity and disease severity. The module contained a total of 98 genes, encoding molecules, such as the S100 proteins S100A8, S100A9, and S100A12, which play a variety of roles in inflammation [3, 13, 21]. Functional annotation clustering indicated that the lightgreen module was enriched for genes involved in classes labeled by GO as ‘immune response’ (*p* = 6.0E-09), ‘negative regulation of cytokine biosynthetic process’ (*p* = 4.3E-03), and ‘regulation of IL-1β production’ (p = 4.3E-03), amongst others (Table 4). These GO classes contained several genes that are directly or indirectly implicated in MS, such as cystatin F (CST7), ghrelin (GHRL), IL-18 receptor accessory protein (IL18RAP), myeloperoxidase (MPO), matrix-metalloproteinase 9 (MMP9), and FMS-like tyrosine-3 (FLT3) [4, 11, 17, 24, 27, 29, 38, 43, 55, 70, 79]. The top 10 genes most strongly connected to the lightgreen module are involved in various molecular mechanisms, such as myelination (CST7), neurogenesis (ASPM), and inflammation (DEFA4, VSTM1, BPI, S100P, WISP3, NLRP12). Overall, the lightgreen module was characterized by molecules that actively regulate inflammation, though it also indicated changes in molecular pathways that affect multiple aspects of MS disease activity to slow down clinical progression.

### A module positively correlated to HPA-axis activity, independent of disease severity

The pink module was positively correlated to cortisol levels (r = 0.56, *p* = 0.003) and numbers of CRH neurons (r = 0.77, *p* < 0.001). The module contained 310 genes and was enriched for molecules with the GO label ‘regulation of caspase activity’ (*p* = 9.4E-04), ‘heat shock protein binding’ (*p* = 8.0E-04), ‘regulation of lymphocyte activation’ (*p* = 2.0E-02), and ‘oxidation reduction’ (*p* = 1.1E-02), amongst others (Table 4). Moreover, the module was enriched for molecules involved in the Kyoto Encyclopedia of Genes and Genomes (KEGG) pathway ‘antigen processing and binding’ (*p* = 1.7E-03). The molecules involved in regulation of caspase activity may be related to apoptosis of neurons and oligodendrocytes, but also play a role in microglia activation and neurotoxicity [7].

Interestingly, the pink module contained the interleukin-7 receptor (IL7R, CD127) and tumor necrosis factor ligand superfamily member 14 (TNFSF14), two molecules that are highly associated with genetic susceptibility to MS [28, 36]. In addition, the pink module contained various heat-shock proteins (HSP), which have been associated with MS pathogenesis, including HSP90, which functions as a chaperone protein for the glucocorticoid receptor (GR) [14, 66]. Also, sirtuin 7 (SRT7) was present in the GO class ‘cellular response to oxidative stress’, which has been described as a protective molecule in the brain and has been proposed as a therapeutic agent [72].

Similar to the observations regarding the lightgreen module, the pink module is also characterized by a combination of genes involved in active regulation of immunity and those involved in more neuroprotective mechanisms.

### Modules negatively correlated to HPA-axis activity, independent of disease severity

The tan module had a module eigengene that was negatively correlated to cortisol levels (*r* = − 0.63, *p* = 7.0E-04) and numbers of CRH neurons (*r* = − 0.61, *p* = 1.0E-03). The module contained 253 genes and was enriched for molecules in the GO class ‘generation of precursor metabolites and energy’ (*p* = 7.6E-10) and ‘mitochondrion’ (*p* = 1.3E-10), which are likely related to changes in mitochondrial functioning and energy metabolism known to occur in MS (Table 4) .

The yellow module had a module eigengene that was strongly correlated negatively to cortisol levels (*r* = − 0.63, *p* = 7.0E-04) and numbers of CRH neurons (*r* = − 0.57, *p* = 3.0E-03). The module contained 922 genes and was enriched for molecules belonging to the GO class ‘lipid biosynthetic process’ (*p* = 1.7E-05) and ‘steroid metabolic process’ (*p* = 2.4E-02) (Table 4). One particularly interesting member of both enriched GO classes in the tan module was aconitase (ACO2). ACO2 is activated by iron and is involved in glutamate production [57], while both iron accumulation and glutamate excitotoxicity are implicated as pathogenic mechanisms in MS [30, 68].

### Genes associated with severity of MS and HPA-axis activity, independent of WGCNA modules

The WGCNA also produces direct correlations between genes and traits of interest, independent of gene modules. These correlations can be used to select individual genes that may be strongly related to HPA-axis activity as well as to disease severity and may therefore offer potential in development of therapeutic strategies for MS. The 10 genes with the strongest positive or negative correlation with disease severity and cortisol are depicted in Table 5.

**Table 5:** Genes most strongly associated to cortisol levels and disease duration

		Cortisol				Duration of MS		
		Gene	Corr	P		Gene	Corr	P
Positive correlations	1	RXRA	0.88	9.1E-09	1	NFE2	0.74	6.8E-04
	2	HEYL	0.83	2.1E-07	2	CHODL	0.72	1.1E-03
	3	PDGFA	0.83	2.9E-07	3	SLC16A6	0.70	1.9E-03
	4	RND3	0.83	3.5E-07	4	DEFB1	0.68	2.5E-03
	5	GJA4	0.82	3.9E-07	5	PSPH	0.68	2.8E-03
	6	IGFBP4	0.82	5.1E-07	6	MYB	0.67	3.2E-03
	7	IL7R	0.81	8.7E-07	7	GDF10	0.67	3.2E-03
	8	NPTX2	0.80	1.3E-06	8	CST7	0.67	3.3E-03
	9	MAOA	0.80	1.8E-06	9	CNTN3	0.67	3.5E-03
	10	TLN2	0.80	1.8E-06	10	HOXA6	0.67	3.6E-03
Negative correlations	1	ANKRD16	−0.82	5.5E-05	1	MSRA	−0.80	1.2E-04
	2	ALKBH3	−0.78	2.1E-04	2	NBR2	−0.71	1.6E-03
	3	DNM2	−0.78	2.5E-04	3	GNG7	−0.67	3.1E-03
	4	TMEM185B	−0.77	2.7E-04	4	SFPQ	−0.67	3.4E-03
	5	FIBP	−0.77	2.8E-04	5	APC	−0.67	3.6E-03
	6	SERAC1	−0.77	3.0E-04	6	SNX8	−0.63	7.0E-03
	7	PLS3	−0.76	3.8E-04	7	COL20A1	−0.61	9.3E-03
	8	ATP5C1	−0.76	4.0E-04	8	CTTNBP2NL	−0.60	1.1E-02
	9	PDE6B	−0.76	4.2E-04	9	RAI14	−0.59	1.2E-02
	10	OPALIN	−0.75	4.9E-04	10	ADAM10	−0.58	1.4E-02

*Corr* correlation coefficient, *P* p-value

Several molecules among the genes positively correlated to cortisol levels are well-known for their prominent role in shaping adaptive immune responses. For example, retinoic acid receptor alpha (RXRA) showed the strongest positive correlation with cortisol levels. Interestingly, RXRA-mediated signaling enhances differentiation of functionally competent CD4^+^ regulatory T cells and potently inhibits the formation of CD4^+^ T helper 17 cells, the latter of which have been strongly implicated in various autoimmune pathologies, including MS [6, 15, 18, 19, 22, 39, 62, 78]. Indeed, retinoic acid and other vitamin-A derivatives were shown to be protective in animal models of autoimmune disease [56]. Notably, IL7R was also present among the 10 genes most positively correlated to cortisol levels. In combination with its presence in the pink module, which showed the strongest positive correlation with cortisol levels in the CSF and numbers of CRH-expressing neurons on the PVN, this further confirms the positive association of IL7R expression with HPA-axis activity in MS. Another interesting members of the 10 genes most positively correlated to cortisol levels is gap junction alpha-4 protein (GJA4), as it is strongly expressed by endothelial cells and may affect leukocyte trafficking [71]. Expression of ankyrin repeat domain-containing protein 16 (ANKRD16) showed the strongest negative correlation to levels of cortisol in CSF. However, not much is known about this protein. The gene most strongly correlated to disease duration is the transcription factor NF-E2 45 kDa subunit (NFE2), which has been shown to play a role in differentiation and maturation of erythroid cells [49]. The strongest negative association to duration was seen for the gene coding for methionine sulphoxide reductase A (MSRA), which is involved in protection against oxidative stress [85].

### Group-wise comparisons using linear regression (LIMMA)

**Fig. 3:**
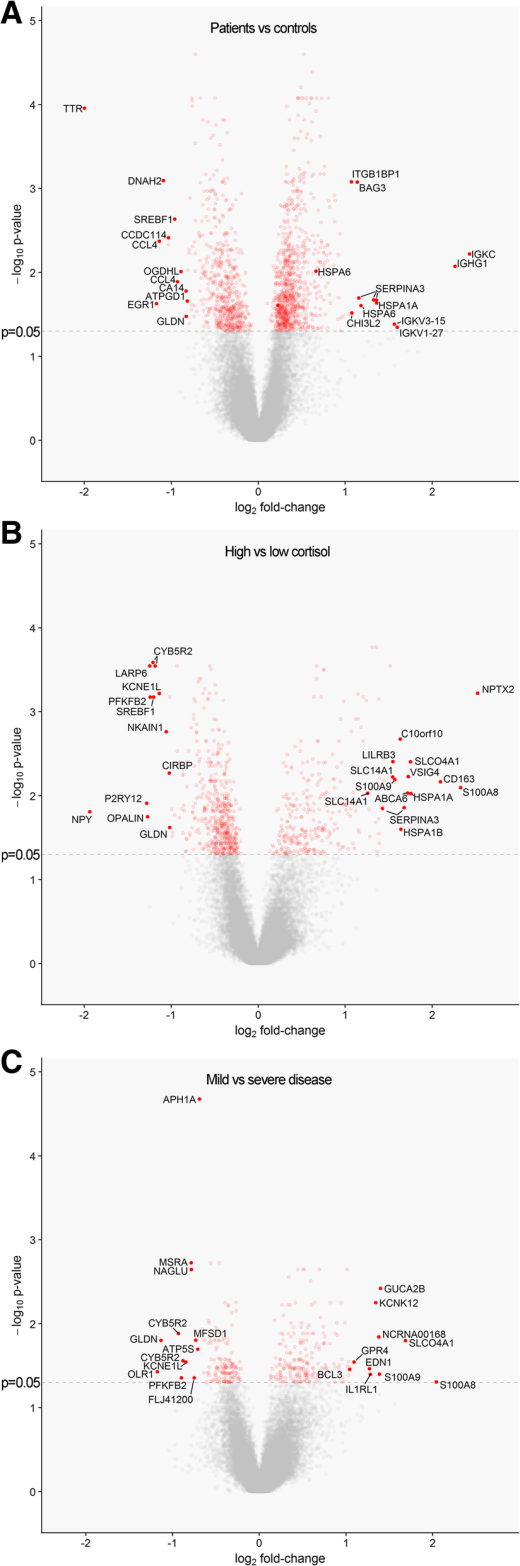
Volcano plots of differential expression between MS donors and controls (a), MS patients with high and low cortisol (b), and MS patients with mild or severe disease (c). Each dot represents one probe on the microarray. Probes with significant differential expression (*p* < 0.05) are plotted in red, all others are grey. Probes representing the 10 most strongly regulated genes in either direction for each comparison are highlighted

The median cortisol level (220.5 nmol/l) was used to objectively divide the MS-patient population into two subgroups, allowing comparison of groups for gene expression profiles that might reveal associations with HPA-axis activity. Similarly, the median duration of MS was used to create an objective subdivision between patients with relatively severe or mild disease (i.e., having a disease course shorter or longer than 24 years, respectively), to compare these groups for identification of differences in gene expression profiles related to disease severity. Two patients in the study population had a disease duration of exactly 24 years. These were included in the group of patients with severe MS, as their disease duration was shorter than the median disease duration of 24.5 years in the entire MS-donor population of the Netherlands Brain Bank. The results of the different comparisons are shown in Fig. 3 and Additional file 2, with the 10 most strongly regulated genes in either direction highlighted. The GO classes enriched in the comparisons according to functional annotation clustering analysis are depicted in Table 6.

**Table 6:** GO classes overrepresented in genes differentially expressed between control subjects and (subgroups of) MS patients

Patients – Controls		
	GO class	Genes present
Up	Positive regulation of apoptosis (GO:0043065)	SIVA1, HTATIP2, ZAK, PML, TNFSF14, RPS27L, TLR4, ITSN1, CTNNBL1, RPS3, CASP3, HTRA2, CD44, CDKN2C, RPS3A, SOS1, MTCH1, TGM2, AATF, RUNX3, RPS27A, DEDD2, CEBPG, FADD, BAD, SOD1, TXNDC12, EI24, RNF7, NAIF1, HSPD1
	Activation of caspase activity (GO:0006919)	SIVA1, MTCH1, PML, HSPE1, HSPD1, RPS3
	Regulation of T cell activation (GO:0050863)	CD47, CASP3, IL6ST, NCK1, TGFBR2, TNFSF14, BAD, HSPD1, SOD1
	Cytokine binding (GO:0019955)	TNFRSF1A, ACVRL1, IL10RB, IL6ST, LEPR, TGFBR2, ENG, IFNAR1, ACVR1
Down	Neuron differentiation (GO:003082)	EGR2, GNAO1, ATL1, TBCE, NTNG2, DSCAML1, APP, RASGRF1, GHRL, MAPK8IP3, SEMA3B, NTM, C17ORF28
	Cell projection (GO:0042995)	RTN4, MYO5A, BBS5, KIAA1598, SSH1, ATL1, NEDD9, GIPC1, DNAH2, CPEB1, APP, CTTN, DNAI1, ARHGEF4, DBNL, ARHGEF7, FSCN1, LDB3, DNAI2, PCM1, CAMK2N1, RASGRF1, IFT172, MAPK8IP3, GHRL, SPEF1
	Regulation of lipid metabolic process (GO:0019216)	TNF, DHCR7, SF1, ACACB, PPARGC1A
MS High Cortisol – Low Cortisol		
	GO class	Genes present
Up	Immune response (GO:0006955)	LAIR1, CEBPB, IL1RL1, IFITM2, TLR2, TNFSF14, CALCOCO2, SLC11A1, C1QB, UNC13D, APOL1, XBP1, LILRB3, IL4R, BCL3, HSPD1, VSIG4
	Regulation of cytokine production (GO:0001817)	INHBA, SLC11A1, CEBPB, TLR2, BCL3, NFKB1, BCL6, HSPD1, VSIG4, SRGN
	Myeloid cell differentiation (GO:0030099)	INHBA, RPS19, JMJD6, RPS14, BCL6, ZBTB16, RUNX1, CBFB, TIMP1
	Negative regulation of myeloid cell differentiation (GO:0045638)	INHBA, NFKBIA, ZBTB16, RUNX1
Down	Apoptosis (GO:0006915)	RTN4, CKAP2, POLR2G, DNM1L, TM2D1, EGLN3, RRAGA, PIGT, BAG1, NGFRAP1, EIF2AK2, MAGEH1, NDUFS1, PUF60, ZIM2
	Negative regulation of neurogenesis (GO:0050768)	RTN4, NOG, NF1, OMG
MS Mild Disease – Severe Disease		
	GO class	Genes present
Up	Neuron projection development (GO:0031175)	APP, GNAO1, EGR2, ATL1, RASGRF1, TBCE, MAPK8IP3, NTNG2, DSCAML1, GHRL, SEMA3B
	Neuron differentiation (GO:0030182)	EGR2, GNAO1, ATL1, TBCE, NTNG2, DSCAML1, APP, RASGRF1, GHRL, MAPK8IP3, SEMA3B, NTM, C17ORF28
	Inflammatory response (GO:0006954)	HDAC5, YWHAZ, TNF, NDST1, TOLLIP, CCL3L3, ITIH4, CCL4L1, CCL4
Down	Lysosome (GO:0005764)	HGSNAT, SGSH, NAGLU, MFSD8, LIPA, GM2A, PPT1, CD63, ASAH1
	Steroid metabolic process (GO:0008202)	HSD17B11, MBTPS2, LIPA, CYB5R2, INSIG2, PRKAA1, CLN6

### Comparison of MS patients and controls

Compared to control subjects, a total of 778 genes was significantly upregulated in MS patients, whereas 544 genes were downregulated. The four most strongly upregulated genes in NAWM of MS patients code for antibody subunits, which is possibly related to the synthesis of auto-antibodies in MS (Fig. 3a) [20]. Furthermore, NAWM of MS patients showed an increased expression of heat-shock proteins, HSPA1A and HSPA6. Among the genes upregulated in NAWM of MS patients compared to that of control subjects, there was an enrichment for molecules in GO classes associated with induction of apoptosis, activation of caspases, regulation of T-cell activation, and cytokines binding (Table 6).

Compared to control NAWM, the gene showing the most strongly decreased expression in MS NAWM was transthyretin (TTR), which was downregulated 4-fold (Fig. 3a). TTR is an important carrier in serum and CSF for the thyroid hormone thyroxin (T4) and for retinol, a form of vitamin A. Importantly, both TTR and T4 have been implicated in MS. Oxidative modifications of TTR protein and decreased levels of T4 were present in the CSF, and not in the serum, of MS patients and were correlated with disease duration [67]. Moreover, T4 was shown to play an important role in activating oligodendrocyte precursor activation and instill myelination [9].

GO classes overrepresented among the genes downregulated in NAWM of MS relative to control NAWM were related to neuron differentiation, cell projection, and regulation of lipid metabolism (Table 6).

### Comparison of MS patients with high and low cortisol

In MS patients with high cortisol a total of 270 genes was upregulated, whereas 472 genes were downregulated compared to patients with low cortisol. Of note, the third most-strongly upregulated gene in MS patients with high cortisol by more than 4-fold was CD163 (Fig. 3b), which is a glucocorticoid-responsive gene that can be induced in myeloid immune cells, such as macrophages and microglia [59, 80].

Neuronal pentraxin-2 (NPTX2) was the gene most strongly increased in NAWM of MS patients with high cortisol, showing an almost 6-fold higher expression in comparison to NAWM of patients with low cortisol (Fig. 3b). NPTX2 is an immune-related molecule with structural similarities to several acute phase proteins that is thought to be essential in the compensatory synaptic response that occurs during prolonged neuronal inactivity [69]. An interesting finding regarding the 10 most strongly downregulated genes in NAWM of MS patients with high cortisol was the presence of the purinergic receptor P2RY12 (Fig. 3b), a microglia signature gene shown to play a major role in microglial activation, synaptic plasticity, and closure of the injured blood-brain barrier [8, 52, 76].

Interestingly, analysis by functional annotation clustering on the genes that are upregulated in MS patients with high cortisol levels, in comparison to patients with low cortisol levels, are enriched for several GO classes associated with (regulation of) inflammation (Table 6). In contrast, there is an overrepresentation of GO classes associated with apoptosis and negative regulation of neurogenesis among the genes showing decreased expression in MS patients with high cortisol.

### Comparison of MS patients with severe and mild disease

In total 202 genes were upregulated in patients with mild MS, whereas 154 genes were downregulated compared to those with severe MS. There is a clear overlap between genes upregulated in MS patients with mild disease and those with high cortisol, as for example S100A8 and solute carrier organic anion transporter family member 4A1 (SLCO4A1) are present in the top 10 most strongly upregulated genes in both groups. IL-1 receptor like 1 (IL1R1) is among the 10 most strongly upregulated genes in mild MS (Fig. 3c), and is known to be a receptor for IL-33 and has been shown to be involved in induction of Th2 responses during allergic inflammation [73]. The oxidized low-density lipoprotein receptor 1 (OLR1) gene, also known as LOX-1, was the most downregulated gene in NAWM of MS patients mild disease (Fig. 3c). Interestingly, this gene was found to be strongly related to the extent of demyelination in white matter MS lesions [33]. The upregulated genes in NAWM of MS patients with mild disease were enriched for GO classes involved in neuron development and differentiation as well as the inflammatory response.

### Distinct expression profile of inflammatory and GC-related genes in patients with high cortisol or mild MS

**Fig. 4:**
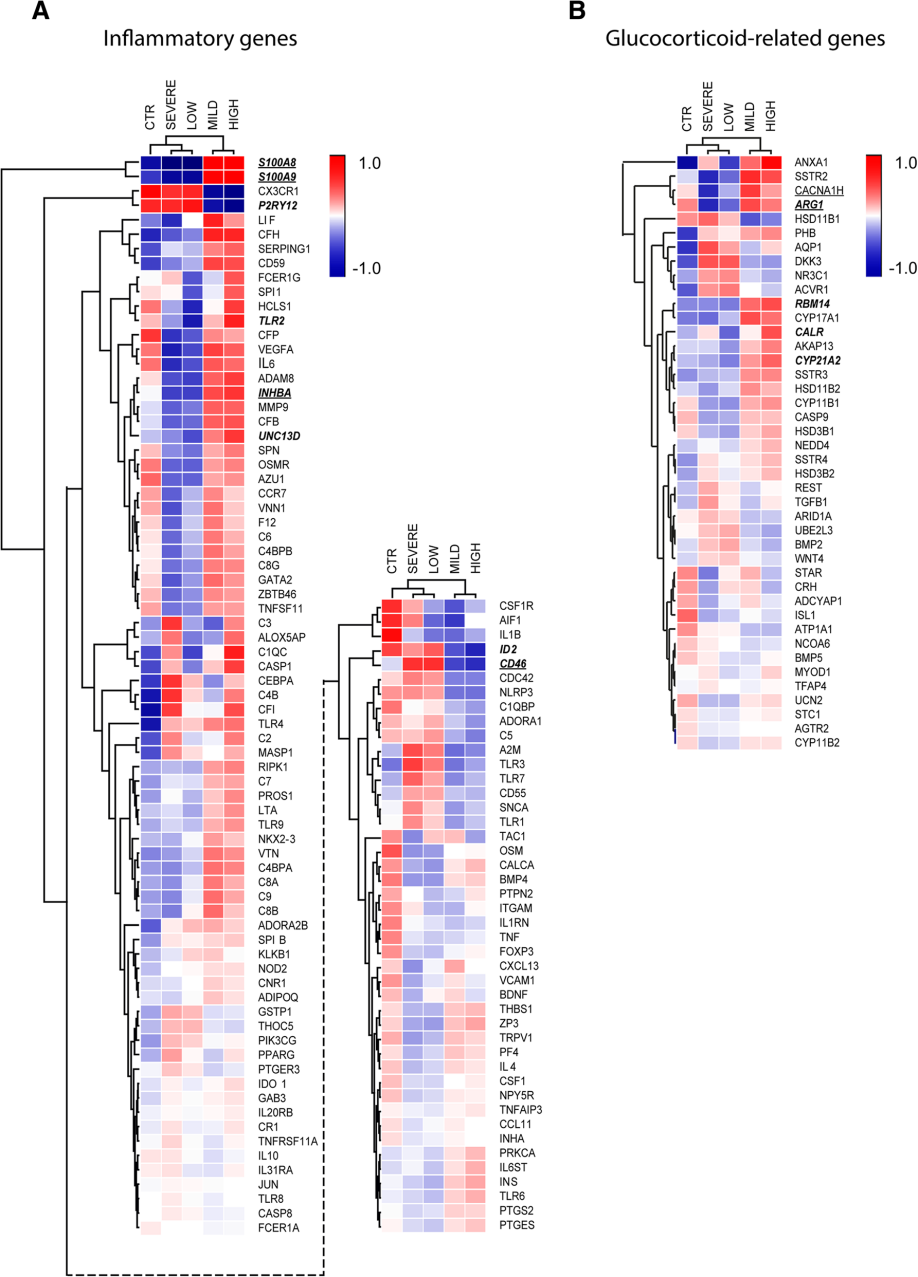
Cluster analysis of absolute expression of genes involved in inflammation and gluco-corticoid signaling. a Cluster analysis based on genes included in the GO classes ‘regulation of acute inflammatory response’, ‘chronic inflammatory response’, ‘macrophage differentiation’, and ‘microglial cell activation’. b Cluster analysis based on genes included in the GO classes ‘glucocorticoid-receptor signaling pathway’, ‘cellular response to glucocorticoids’, and ‘glucocorticoid biosynthetic process’. Underlined are genes for which expression differed significantly between MS patients with mild and severe disease. Genes written in bold and italic show a significant difference between patients with high and low cortisol

For the next analysis, we selected genes included in GO classes related to either inflammation or glucocorticoid signaling, including: ‘regulation of acute inflammatory response’ (GO:0002673), ‘chronic inflammatory response’ (GO:0002544), ‘macrophage differentiation’ (GO:0030225), ‘microglial cell activation’ (GO:0001774), ‘glucocorticoid-receptor signaling pathway’ (GO:0042921), and ‘cellular response to glucocorticoids’ (GO:0071385) and ‘glucocorticoid biosynthetic process’ (GO:0006704). These GO classes were selected to specifically study to what extent HPA-axis activity impacts on molecular mechanisms in NAWM of MS and how this affects neuroinflammation and disease severity. To this end, the selected genes were compared for their expression between five groups: control subjects, MS patients with high and low cortisol levels, and MS patients with severe and mild disease. This was done by cluster analysis, to find out which groups most strongly resemble each other in expression profiles for the selected genes. Interestingly, a distinct expression profile was present for inflammatory and glucocorticoid-associated genes in MS patients with high cortisol and those with mild MS (Fig. 4a and b). These subgroups differed in this respect from MS patients with low cortisol or severe MS, and control subjects. Remarkably, the subgroups of MS patients with high cortisol and those with mild MS were especially similar with respect to higher average expression values for inflammatory genes, when compared to the other three subgroups.

### Molecules selected for validation

**Fig. 5:**
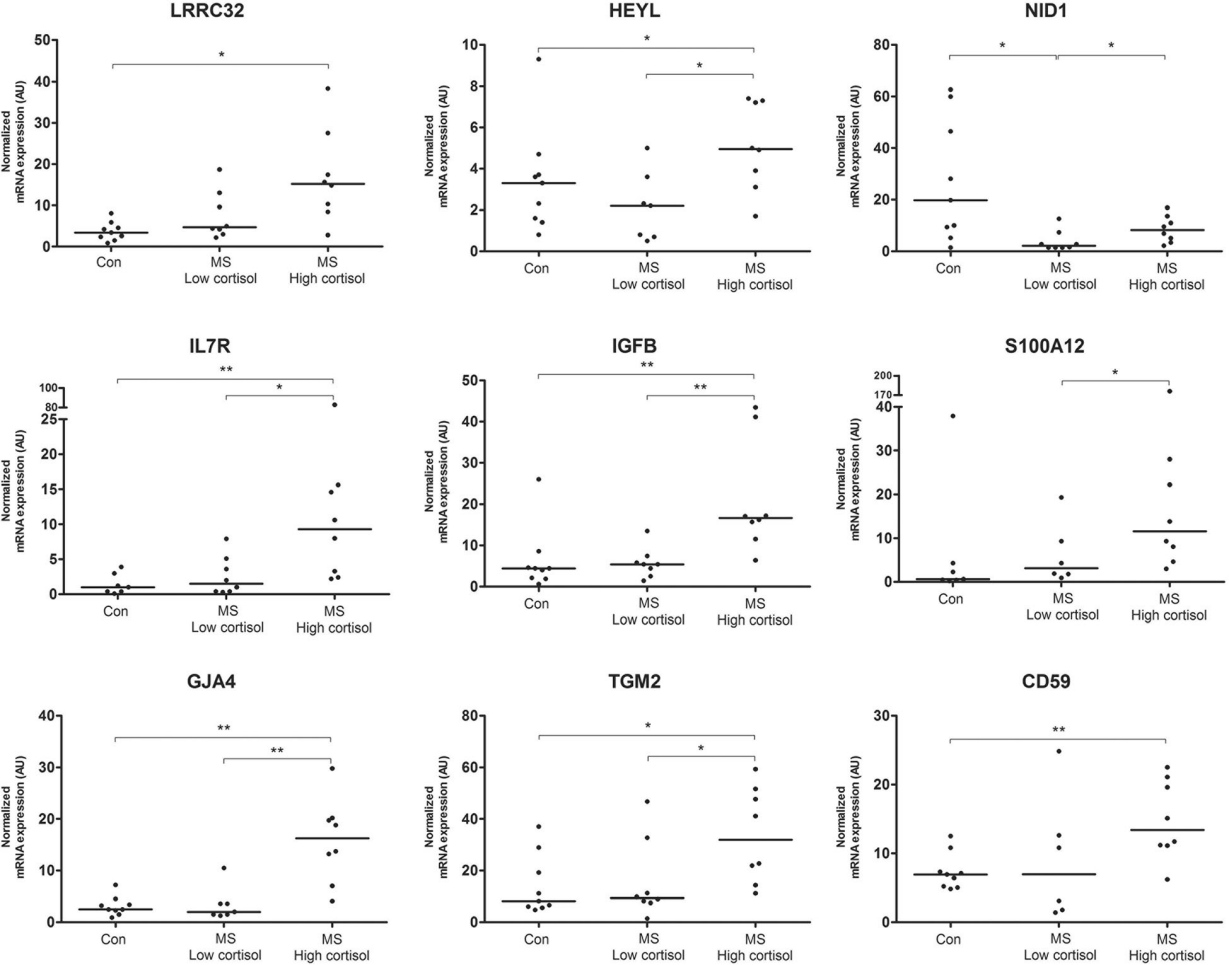
Validation of expression profiles of selected genes by qPCR. Genes were selected for validation on the basis of clear association with HPA-axis activity and/or disease severity in MS patients, as described in detail in the text (Results section). Note that expression of HEYL, IL7R, IGFB, GJA4, and TGM2 is significantly increased in MS patients with high cortisol CSF levels, compared to both control subjects and MS patients with low cortisol. Con = control subjects; AU = arbitrary units; *p < 0.05; **p < 0.001

Expression of in total 14 genes strongly associated with HPA-axis activity and/or disease duration was validated by qPCR. For this purpose, the same tissue was used as for generating the original microarray data. For several of the validated targets no alterations have been described in the context of MS before, such as leucine-rich repeat-containing protein 32 (LRRC32) and nidogen-1 (NID1). Others have been implicated in MS pathogenesis, but were never studied in human subjects for their association with disease severity or HPA-axis activity, such as IL7R, tissue transglutaminase (TGM2), MAC-inhibitory protein (CD59), and gap junction alpha-4 (GJA4) [10, 41, 77, 83]. For 11 out of 14 genes the expression profile was indeed confirmed by qPCR (Fig. 5). Genes for which the qPCR data did not confirm the expression profile detected by microarray were SLC4, CD44, and CST7. For CST7, the expression detected by the microarray as well as the qPCR was very low. Therefore, the expression differences for CST7 observed in the microarray data may have been a technical artifact caused by background variation. For SLC4 and CD44, quite some variation in expression between subgroups was detected by qPCR, but apparently those differences were not large enough to be significant.

## Discussion

This is the very first study, to our knowledge, that reports on genome-wide transcriptional changes in NAWM of MS in relation to disease severity as well as activity of the HPA axis. To this end, we purposefully included female MS brain donors that differed strongly in rate of disease progression and HPA-axis activity. This enabled us to define gene expression profiles associated with disease severity in NAWM that are cortisol-dependent and independent, thereby giving a novel and comprehensive insight into the molecular underpinnings of the clinical heterogeneity that is so characteristic for MS. Importantly, we identified a range of molecular changes associated with a more benign or aggressive disease course in MS that may be targeted for development of new therapeutic strategies. In addition, we uncovered disease-associated gene expression profiles by comparative analysis with white matter from control brain donors, yielding additional molecular targets that may be exploited therapeutically as well.

Data analysis was done using various approaches, including WGCNA and group-wise comparisons of control subjects and (subpopulations of) MS patients. By WGCNA, we were able to identify clusters of co-regulated genes that correlate with one or more indicators of MS-disease severity and HPA-axis activity. Irrespective of their module membership, genes were also studied individually for their association with the same clinical and endocrinological parameters. In this way, the data uncovered many novel genes positively or negatively associated with HPA-axis activity and/or severity of MS. In general, gene expression profiles associated with high cortisol production and mild MS were characterized by molecules that actively regulate inflammation, but also belong to pathways involved in proliferation of neural stem cells. Together, these data reveal that HPA-axis activity strongly impacts on molecular mechanisms/changes in NAWM of MS patients, but these changes are in part independent of the transcriptional changes associated with disease severity.

We show that gene expression profiles associated with high cortisol production and mild MS patients are characterized by molecules that negatively regulate inflammation and immunity, such as NLRP12, S1PR4, S100A8, S100A9, and S100A12 [1, 3, 13, 64, 74]. In this way, our study identifies various molecular targets that may be assessed for their potential to prevent MS pathology in NAWM or limit lesion formation.

The light green gene module is the most relevant module for unraveling the molecular mechanisms of cortisol-mediated suppression of MS-disease progression, as it was correlated to numbers of CRH neurons, cortisol levels, and duration of MS. Functional annotation clustering analysis pointed out that the lightgreen module contained several genes that may be associated with slower progression of MS by regulation of inflammation. Notable molecules present in the lightgreen cluster are S100A8 and S100A9, which are strongly expressed by myeloid immune cells, have well-established immunoregulatory properties and are implicated in protection against oxidative stress [34]. The endogenous danger signal S100A9 plays a key role in immune escape of solid tumors, where its chronic expression in myeloid cells inhibits their maturation and thereby skews them to an immunosuppressive phenotype [13]. Moreover, S100A8 and S100A9 serve as inflammatory biomarkers in several autoimmune disorders, such as systemic lupus erythematosus and inflammatory bowel disease [42, 46, 63]. The anti-inflammatory role of S100A8 and S100A9 are further indicated by the finding that glucocorticoids directly induce these proteins in human monocytes and dendritic cells, and that S100A8-positive macrophages are increased in synovial fluid after treatment of rheumatoid arthritis patients with high-dose methylprednisolone [34].

Another prominent immunoregulatory molecule in the lightgreen module was NLR family, pyrin domain-containing 12 (NLRP12), which was present in several enriched GO classes, such as ‘regulation of IL-1b production’ and ‘negative regulation of cytokine biosynthetic process’. Moreover, NLRP12 expression was highly correlated to cortisol levels. NLRP12 is strongly expressed by myeloid cells and has been found to suppress canonical and non-canonical NF-κB signalling [1, 2, 37, 50, 86]. As such, NLRP12 is able to inhibit Toll-like receptor-induced activation and chemokine production in monocytes and other myeloid cells [50] .

In our WGCNA analysis, we also assessed to what extent the expression pattern of a gene resembles that of the whole module by looking at the connectivity of genes to the module eigengene. Interestingly, GJA4 (gap-junction alpha-4) not only was among the 10 genes most strongly correlated to cortisol, but was also significantly upregulated in mild MS. The expression of GJA4 has been shown to protect against the formation of atherosclerotic plaques by decreased recruitment and local adhesion of monocytes, a mechanism that is thought to be crucially involved in formation of MS lesions [87]. Therefore, high levels of cortisol in MS patients may increase expression of GJA4 on monocytes to diminished their recruitment into the CNS and thereby limit MS pathology.

By studying direct correlations between individual genes with cortisol levels and/or duration of MS, we found several genes that are implicated in (inhibition of) remyelination. Examples are ASPM (abnormal spindle-like microcephaly-associated protein) and TLR2 (Toll-like receptor 2) [31]. Other genes may be more related to neuroprotection. One of these is NPTX2 (neuronal pentraxin-2), which showed a strong correlation to cortisol and was highly increased in MS patients with high cortisol compared to those with low cortisol. This gene is also known as neuronal activity-regulated pentraxin (NARP) and has been shown to be essential for long-term synaptic plasticity, in particular in formation and maintenance of excitatory synapses [69, 88]. As such, NPTX2 may be an important neuroprotective gene in MS that is induced by glucocorticoids.

The pink module showed the strongest positive correlation with HPA-axis activity, both for its correlation with cortisol levels in CSF and numbers of CRH-positive neurons in the PVN. The module was enriched for genes involved in several GO classes, such as ‘regulation of caspase activity’ and ‘heat shock protein binding’. In addition, also genes belonging to the GO class ‘regulation of lymphocyte activation’ were enriched. Among these, the presence of IL7R (IL-7 receptor) was most striking, as it shows a high allelic association with MS susceptibility [28, 41]. IL7R was also found to be induced by dexamethasone in human blood leukocytes, which may explain why its expression was strongly correlated to levels of CSF cortisol in MS patients [23]. Ligation of IL7R by IL-7 was found to be required for autoimmune neuroinflammation in experimental autoimmune encephalomyelitis [47]. Moreover, it was recently reported that downregulation of IL7R expression in oligodendrocytes contributes to CNS demyelination in zebrafish [48]. Thus, IL7R may play opposing roles in MS pathogenesis depending on the cell type it is expressed on. This may also explain why no correlation between IL7R expression and disease severity was observed.

HSPA1A (heat shock protein family A member 1A, Hsp70) and SERPINA3 (serpin family A member 3, alpha1-antichymotrypsin) were higher expressed in NAWM of MS patients with high cortisol compared to those with low cortisol. Heat-shock proteins have been shown to play an important role in limiting T-cell mediated (chronic) inflammation [65, 81]. However, expression of these genes was increased in NAWM of all MS patients when compared to control subjects. This may indicate that upregulation of this pathway represents a general protective mechanism in NAWM of MS that is further enhanced under the influence of high HPA-axis activity.

Importantly, the expression profile of most genes selected for validation could be confirmed by qPCR. However, for the few genes selected for further analysis at the protein level, the signal generated by the antibodies used was not specific enough to enable us to draw clear conclusions about cellullar distribution and potential expression differences. Still, the qPCR data provide valuable biological evidence that observed expression differences in the microarray data are real. In this respect, LRRC32 is particularly notable gene, as qPCR analysis indicated clearly elevated levels of expression in MS patients with high cortisol or mild MS. LRRC32 is a key regulator of transforming growth factor beta (TGF-β), which is known to be involved maintaining the molecular and functional signature of microglia [8]. Moreover, TGF-β is known to promote development of either CD4^+^ regulatory T cells [12]. It is tempting to speculate that, in concert with increased IL7R expression in patients with high cortisol and mild MS, this may indicate the presence of mechanism that serves to maintain a population of T cells with a regulatory phenotype to limit autoimmunity. As the expression of LRRC32 was also very significantly increased in all MS patients compared to controls subjects (data not shown), this could be a general mechanism that is is at play in MS NAWM.

In summary, our data indicate that HPA-axis activity strongly impacts on molecular mechanisms in NAWM of MS patients and thereby has modulatory effects on MS-disease activity. This extends the findings we reported previously [58]. When observing the molecular profiles associated with high HPA-axis activity and relatively mild MS, what is in general most striking is the enhanced expression of genes that actively regulate inflammation. At the same time, NAWM does not show signs of active inflammation. While this has also been indicated by previous studies, our data are the first to indicate more clearly how MS patients may benefit from approaches that promote specific physiological mechanisms for immunosuppression under conditions of chronic inflammation, such as induction of NLRP12, S100A8, and S100A9. At the same time, our data also indicate that high HPA-axis activity is associated with changes in molecular pathways that may affect multiple aspects of MS disease activity to slow down clinical progression. For example, there is a clear increase in the expression of genes involved in remyelination and pathways that likely exert a protective effect against for example oxidative stress, synaptic disintegration, and axonal damage. Most importantly, the study led us to uncover a range of molecular changes associated with a more benign or aggressive disease course in MS that may be targeted for development of new therapeutic strategies. This brings up the important question whether the current regimens for glucocorticoid treatment of MS patients actually promote the molecular mechanisms associated with slow disease progression detected in our study and, if not, how these regimens can be adjusted to ensure that they do. Additionally, it would be important to find out of those same molecular mechanisms associated with slow progression of MS in this study can be induced more effectively or directly without the use of glucocorticoids, especially in those MS patients that respond poorly to glucocorticoids treatment. By comparing MS patients to control subjects, we were able to define disease-associated gene expression profiles in NAWM, which revealed additional molecular targets that may be used for therapeutic exploitation. Since only females were included in this study, it would be important to verify to what extent our findings are also relevant for male patients. This strongly warrants in vitro and in vivo studies to validate which of the molecular targets identified here display the biggest therapeutic potential for the treatment of MS.

## Additional files

Additional file 1: GO analyses. (XLSX 15 kb)

Additional file 2: DE genes. (XLSX 266 kb)
